# Non-native English-speaking applicants and the likelihood of physician assistant program matriculation

**DOI:** 10.1080/10872981.2024.2312713

**Published:** 2024-02-07

**Authors:** Shahpar Najmabadi, Virginia Valentin, Joanne Rolls, Mary Showstark, Leigh Elrod, Carey Barry, Adam Broughton, Michael Bessette, Trenton Honda

**Affiliations:** a Department of Family and Preventive Medicine, University of Utah, Salt Lake City, UT, USA; b Department of PA Studies, College of Health Sciences, University of Kentucky, Lexington, KY, USA; c Affiliate Faculty Yale Institute of Global Health, School of Medicine, Physician Assistant Online Program, Yale University, New Haven, CT, USA; d Department of Medical Sciences, Physician Assistant Program, Northeastern University, Boston, MA, USA; e School of Clinical and Rehabilitation Sciences, Northeastern University, Boston, MA, USA

**Keywords:** ESL, Non-native english-speaking, physician assistant, matriculation, language concordance, multilingual, miscommunication

## Abstract

**Purpose:**

Effective communication is critical in patient care. Multilingual medical providers, including Physician Assistants (PAs) can contribute to improved health care among patients with limited English proficiency; however, this is contingent upon matriculating multilingual providers. In this study, the association between prospective applicants’ self-reported English as second language (ESL) status and their likelihood of matriculation into a PA program was investigated.

**Methods:**

Participants included applicants to five admission cycles of the Centralized Application Service for Physician Assistant from 2012 to 2020. Logistic regression was utilized to investigate association between applicant ESL status and odds of program matriculation in both bivariate and multivariable regression models. Models were adjusted for citizenship status, undergraduate grade point average, gender, age, race/ethnicity, number of programs applied to, and patient care hours.

**Results:**

In unadjusted and adjusted models, ESL status was associated with a significantly lower odds of matriculation to a PA program across all study years. In adjusted multivariable models, associations were strongest for 2014–2015 where ESL status was associated with a 35% lower odds of matriculation (odds ratio 0.65, 95% confidence interval 0.56, 0.76) when controlling for demographics, citizenship status, patient care experience, and academic achievement. In sensitivity analyses restricting to (a) those with TOEFL scores ≥ 100, and (b) restricting to those ESL applicants without TOEFL scores, we did not observe important changes in our results.

**Conclusions:**

Results indicated that non-native English-speaking applicants have lower odds of PA program matriculation. Decrements in matriculation odds were large magnitude, minimally impacted by adjustment for confounders and persistent across the years. These findings suggest that PA program admission processes may disadvantage non-native English-speaking applicants. While there are potential explanations for the observed findings, they are cause for concern. Matriculating and training PAs who have language concordance with underserved populations are important means of improving patient outcomes.

## Introduction

Effective verbal communication between patients and clinicians enhances the likelihood of desired health outcomes in patients from culturally and linguistically diverse backgrounds [1–3]. Language concordance, when a healthcare professional communicates fluently in the patient’s preferred language [2], has a pronounced impact on quality of care and patient safety [1] through preventing adverse events attributed to miscommunication [4]. Miscommunication has been identified as the root cause of 59% of serious adverse events reported to the U.S. Joint Commission’s Sentinel Event Database [1]. Sufficient evidence demonstrates that patients with limited English proficiency (LEP) – defined as self-reported speaking English less than ‘very well’ (i.e., ‘well’, ‘not well’, and ‘not at all’)– [5] are more likely than English-speaking patients to experience safety events because of language barriers [1,6]. Medication errors, readmissions for the same health problem, and prolonged hospital length of stay are common safety risks reported at higher rates among LEP patients [1].

Language is a basic element of culture, including the learned ways of life of different human communities [7]. Patients and providers communicating in the same language is an important aspect of the provision of health care with cultural humility. Language barriers between patients and their clinicians have been linked to several disparities, including less patient-centered care, decreased receipt of recommended preventive health services, diminished joint decision-making, poor patient-clinician communication, and difficulties developing trust [5]. Increased interpreter costs, lower provider and patient satisfaction, and increased opportunities for medical errors are other consequences of language discordance between patients and providers [5]. In addition to undesirable consequences for patients, healthcare professionals may also experience profound adverse psychological effects due to real or perceived errors, the threat of legal action, loss of clinical confidence, and a breach of public trust [4]. Improved communication between patients and their healthcare providers can contribute to a safer and healthier environment for both patients and providers [4].

Currently, there is an ongoing inequity between the diversity of healthcare providers and population trends in the United States [8]. The U.S. foreign-born population reached a record 44.8 million in 2018, and immigrants in 2020 accounted for 13.7% of the U.S. population [9]. By 2055, Asians are projected to become the largest immigrant group in the U.S., and in 2065, those who identify as Asian will make up about 38% of all immigrants; followed by Hispanic, 31%; White, 20%; and Black, 9% [9]. According to the Health Resources and Services Administration (HRSA), American Indian or Alaska Native, Black or African American, Native Hawaiian or other Pacific Islander, and Hispanic (all races) are underrepresented in health professions [10]. However, the population of immigrants is very diverse, beyond the above-listed underrepresented racial/ethnicity population, with almost every country in the world represented among U.S. immigrants [9]. Currently, approximately 20% of Americans speak a language other than English at home and nearly 42% of this group report limited English proficiency. According to the United States Census Bureau, between 1980 and 2019, the increase in people who spoke a language other than English at home (194%) surpassed the increase in the total U.S. population (47%). The current U.S. healthcare workforce lacks the linguistic diversity to address the growing language diversity in the U.S. population. The shortage of multilingual clinicians impairs the growing LEP population’s access to language- and culturally concordant care [11].

Although diversifying medical schools from within, i.e., diversifying the curricula themselves, by providing specific instruction addressing how to work with medical interpreters and/or LEP patients [12], or encouraging U.S. medical students to pursue proficiency in a language besides English [13], can address to some extent the patients and providers language discordance, there is a need for other complementary strategies to catch up with the fast growing language and cultural diversity.

Training multilingual medical providers can directly contribute to improved health care among LEP patients [12,13]. Accordingly, matriculating students with self-reported English as second language (ESL), and foreseeing their educational needs in the curricula can expand diversity among providers and, by extension, improve the care delivered to patients with LEP status. Initiatives to address this much needed language/cultural diversity in the matriculation process of applicants to healthcare professions are largely absent in practice, and notably, there are limited research on ESL students in higher education [14].

The physician assistant (PA) profession is ideally situated to help address this need for language diversity in the healthcare workforce. PAs are highly trained medical professionals, in high-demand by employers, and over 25% of currently practicing PAs work in Primary Care and underserved areas [15]. The diversity of the PA profession does not, however, reflect the diversity of the population that PAs serve in several respects. The vast majority (70.1%) of PAs are women, 80.6% identify as non-Latinx White, and over half are under 40 years of age [15,16]. According to the National Commission on Certification of Physician Assistants (NCCPA) 2021 annual report, the percentage of certified PAs who communicate with patients in languages other than English by the top 10 most frequently identified languages varies between 0.4% (Portuguese, Urdu) to 16.5% (Spanish). Other top 10 languages including Chinese, Russian, French, Hindi, Vietnamese and Arabic is ≤ 1%, and all other languages is 2.2% [15]. PA programs are a rate-limiting step in the development of a PA workforce that could help create a diverse medical workforce to serve the growing non-English proficient population in the United States [17]. Currently, admissions to the U.S. PA programs are very competitive, less than one-third of applicants ultimately matriculate. The application process is complicated, and each PA program across the nation has different application requirements [18–20]. Recent studies indicate that the current matriculation process disadvantage the minorities [16,20–22]. By ensuring equal access to education for students from diverse backgrounds, including applicants with ESL status, PA programs may be able to address this disparity, but it is unknown whether, and to what extent, admissions practices may impact the future workforce of multilingual providers. To address these deficits in the prior literature, we aimed to investigate the association between applicant self-reported ESL status with the odds of matriculation into a PA program.

## Methods

Our methods have been described in detail previously [20]. Briefly, we utilized data from five admission cycles of the Centralized Application Service for Physician Assistant (CASPA), including 2012–2013, 2014–2015, 2016–2017, 2018–2019 and 2020–2021. Over 95% of the accredited national PA programs use CASPA for student recruitment [23]. Participants included all non-duplicate applicants to the respective admissions cycle.

**Figure 1.**
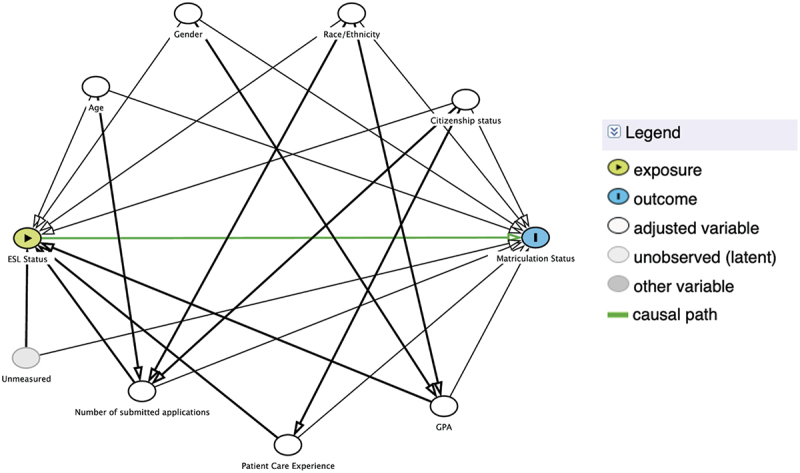
Directed acyclic graph [24] of the conceptual confounding framework in our analysis.

In this study, our outcome of interest was matriculation status (binary, Yes/No) to a U.S. PA program, while our independent variable of interest was self-identified non-native English speaking or ESL status, which was provided by applicants on their CASPA applications (Figure 1).

We utilized logistic regression to investigate an association between applicant self-identified ESL status and odds of program matriculation in both bivariate and multivariable regression models. Models were adjusted for potential confounders, including age, gender, race/ethnicity, citizenship status, number of applications submitted to various programs, patient care experience, and total undergraduate grade point average (GPA) as measure of academic achievement (Figure 1), all identified from prior literature in the field [16,20–22].

Also, in a 3-step sensitivity analysis, we sought to examine whether English proficiency skills were the major drivers of our results. The Internet-based Test of English as a Foreign Language (TOEFL®iBT) is the premier test of academic English communication utilized by U.S. academic institutions. It includes four scaled section scores (reading, listening, speaking, and writing) and a total score. Each section has a score range of 0–30, which are added together for a total score of 0–120. Scores of ≥ 100 are usually used as evidence of substantial English proficiency [25]. Of note, most PA programs require the TOEFL only when other substantial evidence of English proficiency (e.g., graduating from a U.S. undergraduate institution) is missing from the applicant’s profile. To examine non-native English-speaking applicants English proficiency, we restricted non-native English-speaking applicants to those with TOEFL total scores ≥ 100 or an undergraduate or graduate degree from a U.S. accredited academic institution of higher education; in a further step, we restricted non-native English-speaking applicants only to those who met institutional requirements to not report TOEFL scores (e.g., degree from a U.S. institution). Finally, to examine whether TOEFL score among ESL applicants was predictive of matriculation, we further restricted our population to only those with TOEFL scores, and examined the association between TOEFL scores ≥ 100 and < 100 as a binary predictor on program matriculation. Finally, we examined whether race/ethnicity was a modifier of the relationship between ESL status and matriculation.

## Results

**Figure 2.**
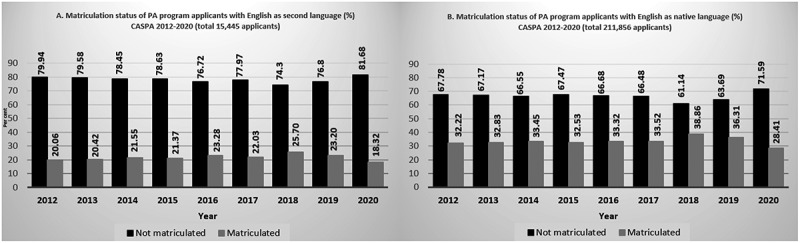
Matriculation status of physician assistant (PA) program applicants by language status per centralized application service for Physician Assistant (CASPA) admission cycle, 2012–2020.

Table 1 summarizes the demographic characteristics of CASPA admission cycle applicants by ESL status over the selected CASPA cycles. Over the past years, total number of CASPA applicants showed a steady increase (up to 1.53 times more from 19,723 applicants in 2012 to 30,123 in 2020). A similar trend (up to 1.62 times more from 1,431 applicants in 2012 to 2,314 in 2020) was also observed in the number of applicants whose self-reported native language was not English. Across the study's lookback period, approximately 6–8% of CASPA applicants identified themselves as non-native English speaking (lowest 5.87% in 2017–2018, and highest 7.68% in 2020–2021). A parallel pattern was observed in matriculation of non-native English-speaking matriculants, with the lowest rate in 2017–2018 (3.94%), with a steadily increase since then, up to 5.09% in 2020–2021. Figure 2(a,b). provide a visual presentation of applicants’ matriculation by ESL status per CASPA admission cycle. As seen, compared to native English-speaking applicants (Figure 2(b)), lower percentage of applicants with ESL status were matriculated over time (Figure 2(a)) (18.32–25.70% versus 28.41–38.86%).

**Table 1.** Demographic characteristics of physician assistant applicants for the selected CASPA cycle years by ESL status [mean (SD) or count (%) ^a^].

		ESL						Non-ESL				
		2012	2014	2016	2018	2020		2012	2014	2016	2018	2020
		1431 (7.26)	1610 (7.14)	1705 (6.38)	1716 (6.40)	2314 (7.68)		18292 (92.74)	20927 (92.86)	25003 (93.62)	25079 (93.60)	27809 (92.32)
**Age**												
Mean (SD)		28.76 (7.54)	28.98 (7.35)	28.67 (7.23)	28.82 (7.43)	28.39 (6.98)		26.03 (6.16)	25.77 (5.84)	25.71 (5.57)	25.50 (5.25)	25.45 (5.35)
Median		26	27	26	26	26		24	24	24	24	24
**Sex**												
Female		1048 (73.24)	1177 (73.11)	1228 (72.02)	1254 (73.08)	1709 (73.85)		13020 (71.18)	14904 (71.22)	17939 (71.75)	18247 (72.76)	20936 (75.28)
Male		382 (26.69)	425 (26.40)	477 (27.98)	461 (26.86)	602 (26.02)		5241(28.65)	5979 (28.57)	7059 (28.23)	6804 (27.13)	6835 (24.58)
Declined		1 (0.07)	5 (0.31)	0 (0.00)	1 (0.06)	3 (0.13)		31 (0.17)	26(0.12)	5 (0.02)	28 (0.11)	38 (0.14)
**Race/Ethnicity**												
White		315 (22.01)	346 (21.49)	317(18.59)	340 (19.81)	397 (17.16)		12760 (69.76)	14299 (68.33)	15593 (62.36)	16010 (63.84)	17500 (62.93)
Black		175 (12.23)	191 (11.86)	204 (11.96)	216 (12.59)	295 (12.75)		1291 (7.06)	1430 (6.83)	1751 (7.00)	2070 (8.25)	2593 (9.32)
Latinx		331 (23.13)	417 (25.90)	499 (29.27)	486(28.32)	752 (32.50)		1346 (7.36)	1668 (7.97)	2198 (8.79)	2551 (10.17)	3099 (11.14)
Asian		451 (31.52)	476 (29.57)	469 (27.51)	480 (27.97)	641 (27.70)		1418 (7.75)	1746 (8.34)	2154 (8.61)	2326 (9.27)	2771 (9.96)
Other^b^		128 (8.94)	154 (9.57)	122 (7.16)	139 (8.10)	174 (7.52)		839 (4.59)	903 (4.31)	1215(4.86)	1209 (4.82)	1256 (4.52)
**U.S. Citizen**												
Yes		1030 (71.98)	1207 (74.97)	1339 (78.53)	1325 (77.21)	1880 (81.24)		17790 (97.26)	20440 (97.67)	24374 (97.48)	24535 (97.83)	27234 (97.93)
No		401 (28.02)	401 (24.91)	366 (21.47)	391 (22.79)	434 (18.76)		492 (2.69)	469 (2.24)	629 (2.52)	544 (2.17)	575 (2.07)
**TOEFL Total Score c**												
TTS <100		-	-	67 (3.93)	70 (4.08)	56 (2.42)		-	-	69 (0.28)	74 (0.30)	440.16
TTS ≥100		-	-	37 (2.17)	26 (1.52)	26 (1.12)		-	-	35 (0.14)	24 (0.10)	180.06
Missing				1601 (93.90)	1620 (94.41)	2232 (96.46)		-	-	24899 (99.58)	24981 (99.61)	27747 (99.78)
Mean (SD)		-	-	93.87 (12.01)	91.68 (13.54)	92.45 (12.03)		-	-	94.17 (12.64)	92.67 (11.41)	91.82 (14.17)
Median		-	-	94.50	93.50	92		-	-	96.50	92	91.50

CASPA: Centralized Application Service for Physician Assistants; SD: Standard Deviation; ESL: English as Second Language.

^a^(%): Column percent, missing not shown

^b^American Indian and Pacific Islander

^c^Ranging from 0–120 (data not available for admission cycles 2012 and 2014; 2016 range (48–117) for total and esl0 49–114 for esl 1, 2018 range (54–116) total and esl0, 61–116 for esl 1, 2020 range (60–117)total and esl0, 70–114 esl1

Overall, ESL applicants were 2–3 years older than native English-speaking applicants. The average age of applicants with ESL status has been steady at 28 years with small fluctuations over time (median 26 with an exception of 27 in 2014), whilst the average age among native English-speaking applicants has slightly decreased over time (from 26.03 years in 2012 to 25.45 years in 2020).

Among applicants with ESL status, the percentage of non-Latinx White applicants was the lowest in 2020 (17.16%). In 2020, Latinx had the highest share of applicants with ESL status (32.50%) followed by Asians (27.70%), Blacks (12.75%) and American Indians/Pacific Islanders (7.52%). For both ESL and non-ESL applicants, more than two-third identified as female across the study period.

The vast majority of non-native English-speaking matriculants did not have a TOEFL total score, indicating that they had a degree from a U.S. accredited academic institute, or were qualified in some other way deemed acceptable to the institutions to which they were applying.

Table 2 shows the results for our bivariate and multivariable logistic regression models. In unadjusted models, ESL status was associated with a significantly lower odds of matriculation to a PA program across all years of our study, with the strongest association observed for 2012–2013, for which ESL status was associated with 47% lower odds of matriculation (95% confidence interval (CI) 40%, 54%) compared to native speakers. In adjusted multivariable models, associations were somewhat attenuated, but remained large magnitude, inverse, and statistically significant across all years, with the strongest association observed for 2014–2015 where ESL status was associated with a 35% decreased odds of matriculation [odds ratio (OR) 0.65 (95% CI 0.56, 0.76)] when controlling for demographics, citizenship status, patient care experience, and academic achievement.

**Table 2.** Association between physician assistant applicants’ ESL status and program matriculation by CASPA cycle year.

	Unadjusted				Adjusted ^a^			
Year	Estimated OR	95% CI		P	Estimated OR	95% CI		P
2012	0.53	0.46	0.60	<.0001	0.73	0.62	0.87	0.0002
2014	0.55	0.48	0.62	<.0001	0.65	0.56	0.76	<.0001
2016	0.61	0.54	0.68	<.0001	0.84	0.73	0.98	0.0218
2018	0.54	0.49	0.61	<.0001	0.78	0.68	0.90	0.0007
2020	0.57	0.51	0.63	<.0001	0.80	0.70	0.91	0.0007

ESL: English Second Language; CASPA: Centralized Application Service for Physician Assistants; OR: Odd Ratio; CI: Confidence Interval.

^a^Adjusted for age at application submission, binary gender, race/ethnicity, application number, citizenship status, hours of patient experience, and cumulative undergraduate total grade point average

**Figure 3.**
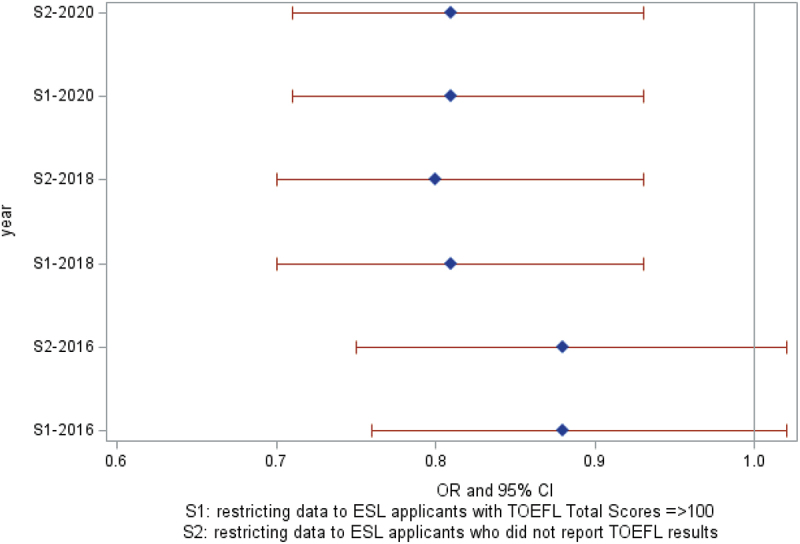
Sensitivity analyses: association between physician assistants’ ESL status and program matriculation by CASPA cycle year.

These findings were not importantly changed with restricting data to ESL applicants with TOEFL total scores ≥100, and in a further step only to those who did not report TOEFL results (Figure 3, Supplemental Table S1).

When restricting the data only to non-English-speaking applicants who had a TOEFL total score, we found lower odds of matriculation for those with scores <100 [2018, OR 0.32 (95% CI 0.12, 0.87), *p*=0.026] which suggests that amongst individuals with TOEFL scores, those with less English proficiency were less likely to matriculate. However, there were small numbers in this restricted dataset (208 applicants in 2016, 194 applicants in 2018, and 144 in 2020) and sparse cell counts, which limited the generalizability and interpretability of these results. Models examining race/ethnicity as a potential effect modifier did not identify statistically significant interaction effects (data not shown).

## Discussion

Our study is the first to suggest that non-native English-speaking candidates have significantly lower odds of PA program matriculation. The decrements in odds were between 20% and 35% in fully adjusted models, minimally impacted by adjustment for important confounders such as age and academic achievement, and persistent across time. What is more, when removing individuals with documented lower English proficiency from the study population by restricting to TOEFL scores ≥100, our findings were not importantly altered. Our study results suggest that PA program admission processes may disadvantage ESL applicants. Despite this potential disadvantage, our study also observed that growth in PA applicants and matriculants is higher among non-native English-speaking students.

The observed growth in ESL applicants is important, as the Pew Research Center projects that by 2050 one in five Americans will be foreign-born [26]. This diverse population will need medical care which ideally will come from a pool of providers who are culturally humble and able to provide excellent care for diverse populations. As diversifying the healthcare workforce is a societal need [27] and a key element to addressing health inequalities [28], educational systems need to contribute to providing all diverse applicants with equal opportunities, which starts at the educational admission process.

The current challenges of medical education have been shaped by systemic racism and exclusionary and segregated education practices [11]. Our study is also timely with the recent supreme court decision limiting the use of affirmative action in admissions practices, purporting that the use of race as a factor in college admissions violated Title VI of the Civil Rights Act of 1964 and the Equal Protection Clause of the Fourteenth Amendment [29]. According to the Title VI, recipients of federal financial assistance are required to take reasonable steps to make their programs, services, and activities accessible by eligible persons with limited English proficiency [30]; and it shaped the opportunity to pass the 1968 Bilingual Education Act (BEA) to articulate the right to a sufficient K-12 education for English learner (EL) students [14]. For implementing language-appropriate care, as required by law, the Culturally and Linguistically Appropriate Services (CLAS) standards recommend either language assistance or a qualified bilingual provider [31]. Currently, language assistance and improving interpreter services have been the main focus in providing language-appropriate care [31]. However, interpreter services increase the cost and duration of treatment [32] and are often complicated by failure to obtain informed consent and protect patient privacy and confidentiality [1]. In addition, the use of interpreter services lead to an increase in adverse events and poorer patient outcomes [32,33], increasing downstream healthcare costs that can be averted by qualified bilingual providers.

Thus, achieving a more equitable future depends on what educational systems do now [11]. English learners right to an education was never extended beyond K-12 education to the higher education level, which is consistent with the notion that postsecondary education is not a guaranteed right for the entire U.S. population [14]. It might also be assumed that an adequate K-12 education would prepare EL students to pursue higher education [14]. However, the disparity between ELs’ and their English-proficient peers’ access to postsecondary institutions remained the same over the past decades [14]. According to Nunez et al., the status of EL policy in K-12 education provides a critical component in understanding the federal and states failure in addressing the needs of EL students in higher education [14]. First and foremost, difficulty arises from lack of specific vocabulary and acronyms used in the professional and academic fields, ranging from terms that may emphasize the lack of English proficiency or, by contrast, the ability to speak another language (limited English proficient vs bilingual) [14]. In this context, we used two old terms, LEP for patients [34] and ESL for the higher education applicants [14]. Second, multilevel intersectionality, i.e., the sociodemographic, sociocultural, and schooling characteristics may significantly limit the number of resources available to ESL students [8,14]. Furthermore, English learners are subject to the effects of local, state and federal policies shaping their academic experiences [14]. All these urges policies to reform the healthcare educational system, not only to adapt the current curricula to encourage native English-speaking students to learn a second language but also to support the matriculation of non-native English-speaking applicants who seek a healthcare degree and meeting their educational needs. Although Title VI and many state laws and adaptive curricula have been in effect for years, still millions of people with LEP are unable to obtain the same quality of care received by English speakers [35]. Adapting policies to reform healthcare education programs’ matriculation processes is an alternative strategy to train PAs and other healthcare workforce with diverse language and cultural background to serve the growing pool of LEP patients. For the PA profession to improve health outcomes, it is critical to recognize the importance of a multilingual health workforce which should begin with support of the ESL student [36].

Currently, PA program admission processes vary by institution [18–20] with programs typically utilizing numerous metrics to evaluate candidates. Several of those metrics that might be impacted by an applicant’s ESL status include evaluations of written essays, review of prior academic achievement, and verbal interviews with candidates who are subject to additional stress for their accent or lack of adequate familiarity to address culturally biased interview questions. Language fluency is an advantage point for native English-speaking applicants. PA program admission processes and committees could also be influenced by implicit or explicit biases against candidates with differing linguistic or cultural backgrounds [8] which necessitates implicit bias training for admissions teams as well as diversifying the admissions teams. Holistic admissions are aimed at optimizing equity in the admissions process and have been described in the medical and PA education literature [19,37–40]. Institutions can take steps to implement the key elements of holistic admissions including (1) broad admissions criteria with connection to excellence through diversity, (2) consideration of academic achievement, characteristics, and experiential background, and (3) individual potential for contributions to the class and the medical field [19,37–40]. With the pressure of accreditation, PA programs may also be concerned that ESL students may require additional program resources or remediation beyond that of their native English-speaking peers.

According to studies performed in Germany, the Netherlands and UK, native speakers as well as bilingual students had significantly better communication skills scores and had a better performance at clinical problem-solving tests than non-native speaking students [27,41,42]. Admission committees and faculty may not be familiar with effective student support strategies and methods for ensuring ESL student success [43]. Some professional graduate programs have addressed this by designing specific programs for ESL learners to improve standardized testing and have shown promising results. Strategies identified in the literature to help ESL students pass the National Physical Therapy Examination and other national licensure examinations included increasing mastery of dialect (English proficiency), enhancing vocabulary development, raising comprehension of questions, and supplementing preparatory activities for at-risk students [43]. Working on vocabulary and associated modifiers has been shown to help ESL students. Foreseeing appropriate accommodations for matriculated ESL students, such as allocating extra time, recording and transcribing lectures, providing audiobooks and library resources in native language, as well as connecting the ESL student to potential mentors/advisors and creating elements of psychological safety can facilitate the adaption process [35]. Rapidly advancing technological resources such as learning management systems (LMS) [44] and mobile assisted language learning (MALL) [45] can be leveraged to support access to PA curriculum for students with LEP. According to Groene et al., non-native speaking students need additional interactive online courses, extra language classes and tailored assessment methods to adjust for the potential additional cognitive load [27,46]. The demonstrated effectiveness of social media in sharing medical education materials such as YouTube, to the medical literature review, using a learning form commonly referred to as ‘Free Open Access Medical Education’ in low‑resource settings [47] may be a viable strategy for improving English language proficiency among non-native English-speaking students matriculated to a PA-program. Creation of a non-native native English-speaking PA-students collaborative medical education Facebook page is also a possible solution to the problem [47].

The emphasis on barriers to matriculation of ESL applicants should be balanced with promoting protective factors [8]. Despite specific challenges of linguistic capability of ESL students and differences in cultural expectations of the role of learners and teachers in postgraduate studies, this is also an opportunity. It brings benefits to both non-native and native English-speaking students and professors as they learn more about each other and come to understand differences and build on aspects they have in common [48], as well improving the cultural competency of the PA cohort. Furthermore, using peer assisted learning can improve both native and non-native English-speaking students’ comfort in communicating with LEP patients. The feasible, low-cost program using peer assisted learning has been a successful strategy for medical students in the United Arab Emirates, who is due to using English as the language of instruction, lack confidence and feel unprepared to communicate effectively with the local population [2].

As institutions look to address the future health workforce needs that are compliant with the law, language concordance could be an important factor. The U.S. census bureau’s primary purpose of collecting language data is to measure the proportion of the U.S. population that may need help in English comprehension [49]. According to the language data, English-speaking ability is a major indicator of educational attainment and poverty level [49], altogether lead to health disparity and poor health outcomes. Providers/patients language concordance is one of the feasible solution to address the health needs of non-native English-speaking patients. Recruitment and meeting the educational needs of disadvantaged non-English-speaking applicants to healthcare-related professions is one of the available solutions. To allocate healthcare resources including training healthcare workforce thoughtfully, medical schools and all other healthcare-related professions can benefit the language data in their students’ recruitment policy.

### Study limitations

Our study has a number of limitations, which may suggest areas for future research. While we found little differences in association when excluding those with low TOEFL scores (in an attempt to exclude those with significantly limited English proficiency), we also observed that the vast majority of ESL students did not have TOEFL scores, which indicates they likely qualified for admission by graduating from a U.S. institution (or in some cases another country with English as a primary language). It is possible, though, that this non-TOEFL qualification does not robustly identify individuals with severely limited English proficiency, and that these limitations only become known to the programs during the application and interview processes. This is supported by our findings that low TOEFL scores are associated with lower likelihood of matriculation when examining only ESL students with TOEFL scores. This may indicate that the TOEFL, while helpful, is systematically underutilized by institutions who rely more on a prior degree from an English-speaking institution. Unfortunately, our dataset does not allow us to discern this. Other limitations include that all data are self-reported, and that a large majority, but not all PA programs use CASPA, and thus our findings are not universally transportable. Furthermore, although CASPA collects data on applicants' spoken language, what’s not clear in the application is whether someone has two first languages – such as someone who grew up speaking English plus another language, which is common, so not all are ESL, per se [15,50]. Also, knowing whether a student had spoken English as a first language, does not distinguish between lower proficiency English learner applicants and English-proficient English learner applicants [14]. Future research should investigate the educational outcomes and support needed for ESL students to be successful in these rigorous health profession programs.

### Conclusions

As the U.S. nation remains to be a new home for people from other countries, and as different groups maintain their use of language as a hallmark of their cultural identity [7], the current pattern of language diversity may very well continue [49]. In the past when racial bias has been addressed in admission processes via a number of strategies, the percent of underrepresented students increases and standardized test scores have maintained or improved [51]. Removing the structural bias faced by medical or associated fields’ applicants who are not native English speakers and addressing their specific educational needs will not only help to address career development difficulties faced by ESL applicants, it also fills the gap in providers/patients language and cultural discordance. Currently, non-native English speakers’ effort and investment in improving their English skills is required for overcoming language barriers. However, the disadvantage magnitude is far beyond the level that can overcome with individuals’ efforts [52]. As all institutions re-evaluate admission processes to assure compliance with the recent supreme court decision to limit affirmative action, language barrier is an important factor to reconsider.

## Supplementary Material

Supplemental Material

## Data Availability

Data are available from authors upon reasonable request, subject to PAEA and institutional and research ethics (IRB) approval.
