# Supplementary material for: Non-native English-speaking applicants and the likelihood of physician assistant program matriculation
Source: Med Educ Online. 2024 Feb 7;29(1):2312713. doi: 10.1080/10872981.2024.2312713 (PMC10851801; doi:10.1080/10872981.2024.2312713)
Supplement: Supplemental Material [file ZMEO_A_2312713_SM2994.docx]

**Supplemental Table 1.** Sensitivity Analyses: Association between physician assistant applicants’ ESL status and program matriculation by CASPA cycle year

| **Year** |  | **Adjusted**^a^ **Estimated OR** | **95% CI** | | **P** |
| --- | --- | --- | --- | --- | --- |
|  |  |  |  |  |  |
| **Sensitivity Analysis 1: Excluded applicants with a TOEFL Total Score <100** | | | | | |
|  |  |  |  |  |  |
| **2016** |  | 0.88 | 0.76 | 1.02 | 0.0922 |
|  |  |  |  |  |  |
| **2018** |  | 0.81 | 0.70 | 0.93 | 0.0037 |
|  |  |  |  |  |  |
| **2020** |  | 0.81 | 0.71 | 0.93 | 0.0025 |
|  |  |  |  |  |  |
| **Sensitivity Analysis 2: Excluded all applicants with a TOEFL Total Score** | | | | | |
|  |  |  |  |  |  |
| **2016** |  | 0.88 | 0.75 | 1.02 | 0.0895 |
|  |  |  |  |  |  |
| **2018** |  | 0.80 | 0.70 | 0.93 | 0.0037 |
|  |  |  |  |  |  |
| **2020** |  | 0.81 | 0.71 | 0.93 | 0.0021 |
|  |  |  |  |  |  |

ESL: English Second Language; CASPA: Centralized Application Service for Physician Assistants; OR: Odd Ratio; CI: Confidence Interval

^a^ Adjusted for age at application submission, binary gender, race/ethnicity, application number, citizenship status, hours of patient experience, and cumulative undergraduate total grade point average

**Supplemental Table 2.** Assessment of the importance of the controlled covariates (combined CASPA cycle year 2012-2020)

| **Contrast Estimate Results - 2012-2020 combined data** | | | | | |  |
| --- | --- | --- | --- | --- | --- | --- |
| **Label** | **L'Beta Estimate** | **Standard**  **Error** | **L'Beta**  **Confidence Limits** | | **Pr >ChiSq** |  |
|  |  |  |  | |  |  |
| **Unadjusted** | | | | | |  |
| **Exp(Beta ESL vs native)** | 0.5493 | 0.011 | 0.5281 | 0.5713 | <.0001 |  |
| **Adjusted** | | | | | | **Covariates added one at a time**^a^ |
| **Exp(Beta ESL vs native)** | 0.5493 | 0.011 | 0.5281 | 0.5713 | <.0001 | Gender |
| **Exp(Beta ESL vs native)** | 0.5601 | 0.0114 | 0.5381 | 0.5829 | <.0001 | Number of applications |
| **Exp(Beta ESL vs native)** | 0.5955 | 0.0332 | 0.5338 | 0.6643 | <.0001 | Patient experience (hour) |
| **Exp(Beta ESL vs native)** | 0.5958 | 0.0123 | 0.5722 | 0.6203 | <.0001 | Citizenship status |
| **Exp(Beta ESL vs native)** | 0.6169 | 0.0137 | 0.5906 | 0.6444 | <.0001 | GPA |
| **Exp(Beta ESL vs native)** | 0.6316 | 0.0129 | 0.6069 | 0.6573 | <.0001 | Age |
| **Exp(Beta ESL vs native)** | 0.6762 | 0.0143 | 0.6486 | 0.7049 | <.0001 | Race |

^a^ Except gender, from top to the bottom the effect of ESL status slightly was attenuated when adjusted for each of these individual covariates.
